# Unwanted acquired mutations in Ba/F3 transformation assays

**DOI:** 10.18632/oncotarget.16268

**Published:** 2017-03-16

**Authors:** Jean-Baptiste Demoulin,, Guillaume Dachy, Florence A. Arts

**Affiliations:** De Duve Institute, Universite catholique de Louvain,Brussels,Belgium

**Keywords:** kinase, oncogene, signaling, cytokine receptor

Cancer genome sequencing has unraveled the huge variety of DNA alterations acquired during cell transformation. Only a small part of these alterations are thought to promote cancer development, while others represent mere random passenger mutations. It is essential to decipher which mutations are driving the oncogenic process to translate cancer sequencing data into clinically meaningful information, which can be used for precision medicine. Prediction software, such as Polyphen or MutationTaster, almost instantly give a useful estimate of the probability that a newly-identified variant affects the corresponding gene function. Nevertheless, experimental work is still required to definitively establish the importance of each mutation [1]. In this respect, anum ber of cell-based assays are available to test the functional impact of newly discovered mutations. In hematology, one of the most popular transformation assays is based on Ba/F3 cells and was first described in 1988 by George Daley and David Baltimore to demonstrate the oncogenic potential of BCR-ABL [2]. Ba/F3 cells have been subsequently used in more than one thousand publications referenced in PubMed.

**Figure 1:**
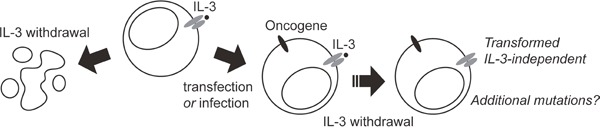
The Ba/F3 cell transformation process

The Ba/F3 cell line was isolated from mouse normal bone marrow cells cultured in the presence of interleukin-3 (IL-3) [3]. These cells undergo massive apoptosis as soon as IL-3 is removed from the culture medium. However, Ba!F3 cells that are engineered to express an oncogene, such as BCR-ABL or a mutant receptor, can proliferate indefinitely in the absence ofiL-3 (Figure 1). These cells are considered as transformed, because, unlike parental cells, they induce leukemia when reinjected in syngeneic Balb/c or immunodeficient mice [4, 5]. One key advantage of the Ba!F3 model is the absence of spontaneous transformation of untransfected cells.

After Ba/F3 transfection, only a fraction of the cells expressing the oncogene adapt to IL-3-independent proliferation. The selection of these cells in culture takes a few days to a few weeks, depending on the potency of the tested oncogene. The molecular events underlying this selection process have remained unclear for many years. In this issue of Oncotarget, Kevin Watanabe-Smith *et aL* [6] show that spontaneous additional mutations arise in some oncogenic transgenes during the adaptation to IL-3 independence. Strikingly, they also observe that a wild-type receptor transgene, CSF3R, also acquires mutations and transforms Ba/F3 cells. This does not occur with empty control vectors, in agreement with previous publications. This report is of critical importance to the community of researchers who use Ba!F3 cells to study mutants and might have to revise the conclusions of some of their experiments. The publication of Daley and Baltimore is not at stake here: Watanabe-Smith and colleagues did not detect any additional mutation in this potent oncogene after transduction in Ba/F3 cells. Instead, unexpected acquired mutations occur in weakly transforming constructs, which require longer periods of time to transform cells. One problem is that many publications do not provide information regarding the potency of the tested transforming transgene and the efficiency of the Ba!F3 selection step. Instead, a large part of the scientific literature is based on experiments performed on cells that are fully transformed and have been growing in the absence of IL-3 for quite some time.

The authors also studied the mechanism whereby transgenes acquired extra mutations. They first ruled out the possibility that widespread genomic instability occurs in transduced Ba/F3 cells. They also showed that the mutations were already present in a small proportion of cells before IL-3 withdrawal, suggesting that the culprit may be the error-prone reverse transcriptase, during the production of the retroviruses used to infect Ba/F3 cells. The vector integration into the cell genome could also be at the origin of some of the observed transgene alterations, such as large deletions. Future studies should determine whether the rate of unwanted mutations may be lowered by using alternative transfection strategies.

Based on these striking results, the authors make two important recommendations to improve Ba/F3 results reliability: (1) to sequence the transgene after transformation and (2) to provide quantitative data on the transgene potency. The latter recommendation can be relatively easily met by calculating time to outgrowth, as an indicator of the period of time required to produce fully transformed IL-3-independent cells. Alternatively, the rate of transformation can be calculated from limiting dilution experiments, which are more cumbersome but may provide a better readout.

Ba/F3 cells expressing transforming oncogenes have been extensively used as a bioassay to test targeted therapies, such as tyrosine kinase inhibitors, as illustrated by our own work [1, 5, 7]. Obviously, the presence of additional unwanted mutations in the transgene may dramatically alter drugs sensitivity. In these assays, sequencing the transgene should become a standard practice. This may not be enough, however, as alterations in transformed Ba!F3 genomes may not be restricted to the transgene: Hornakova and colleagues observed that Ba!F3 cells expressing a mutant IL-9 receptor acquired activating mutations in the endogenous *Jakl* gene during the transformation process [7]. Sequencing the whole genome of transformed Ba!F3 cell lines might be one way to search for such additional mutations, but the interpretation of Ba/F3 genomic data and its variations is challenging. The work of Watanabe-Smith suggests a simple solution to this issue: using several independent Ba!F3 cell lines may circumvent the problem because acquired mutations appear to be highly variable, random and non-recurrent. So this brings us back to the one of the most important scientific recommendation of our mentors and supervisors: always perform biological replicates of your experiments.
